# Rapid seedling emergence of invasive *Phytolacca americana* is related to higher soluble sugars produced by starch metabolism and photosynthesis compared to native *P. acinosa*

**DOI:** 10.3389/fpls.2024.1255698

**Published:** 2024-01-26

**Authors:** Danfeng Liu, Maoye Liu, Ruiting Ju, Bo Li, Yi Wang

**Affiliations:** ^1^ Ministry of Education Key Laboratory for Transboundary Ecosecurity of Southwest China, Yunnan Key Laboratory of Plant Reproductive Adaptation and Evolutionary Ecology and Centre for Invasion Biology, Institute of Biodiversity, School of Ecology and Environmental Science, Yunnan University, Kunming, China; ^2^ Ministry of Education Key Laboratory for Biodiversity Science and Ecological Engineering, Institute of Biodiversity Science, Fudan University, Shanghai, China

**Keywords:** rapid seedling emergence, invasive *Phytolacca americana*, β-amylase, photosynthesis, soluble sugar

## Abstract

Seedling emergence is an essential event in the life cycle of plants. Most invasive plants have an advantage in population colonization over native congeners. However, differential seedling emergence between invasive plants and native congeners, especially their mechanisms, have rarely been explored. In this study, we show that the seedlings of invasive *Phytolacca americana* emerge faster compared to native *P. acinosa*. Genome-wide transcriptomes of initially germinated seeds versus seedlings at 4 days after germination (DAG) suggested that differentially expressed genes (DEGs) in the photosynthesis-antenna proteins pathway were up-regulated in both *P. americana* and *P. acinosa*, while DEGs in starch and sucrose metabolism were significantly down-regulated in *P. americana*. Gene expression analysis indicated that photosynthesis-related DEGs reached their highest level at 3 DAG in *P. americana*, while they peaked at 4 DAG in *P. acinosa*. We also identified one β-amylase gene in *P. americana* (*PameAMYB*) that showed the highest expression at 1 DAG, and two β-amylase genes in *P. acinosa* that expressed lower than *PameAMYB* at 0 and 1 DAG. Enzymatic activity of β-amylases also suggested that *P. americana* had the highest activity at 1 DAG, which was earlier than *P. acinosa* (at 4 DAG). Soluble sugars, the main source of energy for seedling emergence, were showed higher in *P. americana* than in *P. acinosa*, and reached the highest at 4 DAG that positively affected by photosynthesis. These results indicate that the rapid seedling emergence of invasive *P. americana* benefited from the high soluble sugar content produced by starch metabolism and photosynthesis. Altogether, this work contributes to our fundamental knowledge on physiological and molecular mechanisms for plant invasion success.

## Introduction

Seedling emergence occurs after seed germination, when the shoot and root of the seed are formed (Csontos, 2003), and is an essential prerequisite for vegetative growth, population colonization, and dispersal of plants (Verdú and Traveset, 2005; Leger et al., 2019; Larson et al., 2020). However, emerging seedlings are vulnerable to many extrinsic factors, such as water, pests, and maternal effects (Lamichhane et al., 2018; Chen et al., 2020). Seed-to-seedling transition is also affected by intrinsic factors, including plant genotypes (Graebner et al., 2012; Souza and Fagundes, 2014; Yu et al., 2020). Therefore, short time intervals between seed germination and seedling emergence are usually helpful for successful establishment of seedlings.

Seedling emergence is highly dependent on plant physiological status. Typically, soluble sugars are the main source of energy for seedling emergence (Penfield et al., 2005). Hydrolytic enzymes, including α-amylase, β-amylase, and α-glucosidase, are the primary enzymes that break down starch into soluble sugars (Zeeman et al., 2010). In *Triticum aestivum*, post-germination seedling growth is positively regulated by starch degradation that is catalyzed by α-amylase and α-glucosidase (Sun et al., 2020). The β-amylase activity in *Cyclobalanopsis gilva* was found to be higher in germinated seeds compared to non-germinated seeds (Zaynab et al., 2018). Therefore, high hydrolytic enzyme activity in germinated seeds is crucial for rapid seedling emergence.

Invasive plants cause serious ecological consequences and pose great threats to the economy and human health (Rands et al., 2010; Vila et al., 2011; Rai and Singh, 2020). Most invasive plants grow more rapidly than their native congeners, such as *Copaifera langsdorffii* versus *C. oblongifolia*, *Spartina densiflora* versus *S. maritime*, as well as *Phytolacca americana* versus *P. acinosa* (Fagundes et al., 2020; Infante-Izquierdo et al., 2020; Liu et al., 2022). Functional traits of plants, like specific leaf area and photosynthetic rate, have been an active point of research on the rapidity of plant growth (Feng et al., 2009; Liu et al., 2022). However, the mechanisms that underlie the physiological and molecular differences of plant growth between invasive and native congeners are still poorly understood, especially during the transition from seeds to seedlings.

*P. americana* is an herbaceous perennial plant native to North America that occupies a wide range of habitats in China, and is known to be poisonous to mammals (Xu et al., 2012). Meanwhile, *P. acinosa* is the congener of *P. americana* that is native to China. In this study, we compared the differences in seedling emergence of invasive *P. americana* and native *P. acinosa*. Firstly, the number of emerged seedlings for the two species was recorded in the 10 days after seed germination (DAG). Subsequently, genome-wide transcriptomes were sequenced for emerged seedlings at 0 and 4 DAG for both species. According to the differential metabolic pathways between *P. americana* and *P. acinosa*, photosynthesis-related differentially expressed genes (DEGs) and differentially expressed β-amylase genes were further investigated. Additionally, gene expression and enzymatic activity of β-amylase, and total soluble sugar content were determined to investigate the effect of photosynthesis and starch metabolism on seedling emergence. These results would help illustrate the physiological and molecular mechanisms of differential seedling emergence between the two congeners, and further reveal the invasion mechanisms of *P. americana*.

## Materials and methods

### Plant materials

Seeds of *P. americana* (24°49′ N, 102°52′ E) and *P. acinosa* (25°26′ N, 104°19′ E) were collected from the field in August 2020 in Yunnan, China. Mature racemes, that grew under adequate sunlight in the canopy, were randomly collected from each plant species. Seeds were obtained after the raceme flesh were removed (Liu D. et al., 2020). All seeds were stored in the laboratory at the Institute of Biodiversity, School of Ecology and Environmental Science, Yunnan University. The 1,000-seed weight of *P. americana* and *P. acinosa* was recorded as 7.55 ± 0.08 g and 7.56 ± 0.03 g, respectively. For germination, all seeds were soaked in 98% H_2_SO_4_ for 15 min to break dormancy. Subsequently, seeds were separately placed in Petri dishes (d = 9 cm) with 20 mL of 1% agar medium (1g agar and 100 mL ddH_2_O) and cultured in a growth chamber (Yiheng, Shanghai, China) with a 14 h light: 10 h dark cycle at 27°C and 25°C, respectively.

### Germination rate, seedling emergence, and seedling biomass measurement

To compare the difference in time intervals between seed germination and seedling emergence, we first calculated seed germination rate of *P. americana* and *P. acinosa*. Six seeds of one species were placed in a Petri dish with agar medium and cultured as described above. Ten replicates were performed. The seeds were considered to have germinated when approximately 1 mm of the radicle protruded from the seed coat. At day 3, the radicle of the seed emerged, and the number of germinated seeds was recorded. The germination rate was determined as the number of germinated seeds at day 3 versus the total number of tested seeds.

The seeds that germinated at day 3 were employed for seedling emergence tests and treated as 0 DAG. Twenty initially germinated seeds of *P. americana* and *P. acinosa* were separately transferred into another 1% agar medium and cultured as described above. Three replicates were conducted. Seedlings with spread cotyledons were used for seedling emergence analysis (Larson et al., 2020). The number of emerged seedlings was recorded daily for 10 days. At 10 DAG, the fresh weight of the seedlings was measured using an analytical balance (BSA223S, OLABO, Shandong, China).

### RNA isolation, Illumina sequencing, and data processing

Seedlings at 4 DAG of each species were individually collected for transcriptome analysis, and 0 DAG seedlings were used as controls (**Supplementary Figure S1**). Eight germinated seeds were used for one replicate, and three biological replicates were conducted. Total RNA was extracted using the RNAprep Pure Plant Kit (Tiangen Biotech Co., Ltd, Beijing, China) following the manufacturer’s instructions. RNA quality and purity were determined using a NanoPhotometer N60 (Implen, Germany) and an Agilent Bioanalyzer 2100 system (Agilent Technologies, CA, USA). A sample of 1 μg RNA was used, and cDNA libraries were constructed separately, as previously described (Liu D. et al., 2020). Transcripts at the genome-wide transcriptional level were separately determined using pair-end (150 bp) RNA sequencing (RNA-Seq) on an Illumina NovaSeq 6000 platform at Biomarker Technology Co. (Beijing, China).

Clean reads were obtained by removing adaptor, reads with Q30 (probability of incorrect base call = 1/1000) of less than 85%, and reads containing more than 10% unknown bases per read from raw reads. After trimming, transcripts were assembled by pooling clean reads for all individual plant treatments using Trinity (v2.5.1), with min_kmer_cov set to 2 (Grabherr et al., 2011). To quantify gene expression, clean reads were mapped back to the transcriptome dataset, and read numbers were obtained using RSEM (v1.2.19; Li and Dewey, 2011). The mapped reads were normalized according to the fragment per kilobase of exon model per million mapped reads (FPKM; Mortazavi et al., 2008). Transcripts with a *P*-adjusted (*P*-adj) value of < 0.05 between two groups (each group with three biological replicates) and absolute log_2_ (0 DAG verse 4 DAG) > 1 were assigned as DEGs (Anders and Huber, 2010; Wu et al., 2021). Gene ontology (GO) enrichment analysis of the DEGs was performed using the topGO R package (v2.28.0) based on the Kolmogorov-Smirnov test (Alexa and Rahnehfuhrer, 2016). KOBAS2.0 software was used to test the statistical enrichment of DEGs in the Kyoto Encyclopedia of Genes and Genomes (KEGG) pathways (Xie et al., 2011). Raw data were deposited in the Sequence Read Archive of the National Center for Biotechnology Information (accession number: PRJNA992375).

### Candidate gene selection

We selected DEGs from KEGG pathways with *P* < 0.05 between the two developmental stages, and DEGs with FPKM value > 10 and log_2_foldchange (0 DAG versus 4 DAG) > 2 or < -2 in each pathway were filtered and then analyzed in R (4.1.3, R Core Development Team, 2020) using the GOplot package.

We found that DEGs enriched in the photosynthesis-antenna proteins pathway were up-regulated in both *P. americana* and *P. acinosa*. To better analyze the phylogenetic relationship among these DEGs, we constructed a phylogenetic tree according to their deduced amino acid sequences with MEGA 11.0 using the Neighbor-Joining method with 1000 bootstrap replicates (Hall and Notes, 2013). FPKM values for the DEGs were also presented using iTOL (https://itol.embl.de/). To further analyze gene expression, some homologous DEGs between the two species were randomly selected. These genes were amplified with the primers listed in **Table 1** and cloned into a sequencing vector BluntSimple (TransGen, China) for full sequencing. Quantitative polymerase chain reaction (qPCR) was then performed to determine the gene expression of the seedlings at 0, 1, 2, 3, and 4 DAG.

**Table 1:** Primers for genes involved in photosynthesis-antenna proteins pathway.

Name	Sequences 5’-3’
For sequence amplification	
EVM0007929F	ATGAGGAAGACTGCAGCCAAA
EVM0007929R	TTACTTGCCAGGGACAAAG
EVM0015298F	ATGGCTTCCTCTACAATGGCA
EVM0015298R	TCACTTGCCGGGAACAAAGTTT
EVM0020215F	ATGGCAACAATGTTGAGCTC
EVM0020215R	TTATGAGCCAGGCACAAAC
EVM0004062F	ATGGCTGCATCTACAATCCAA
EVM0004062R	TCACTTTCCAGGGACAAAGTT
Pac02G011390F	ATGGCTTCCTCTACAATGGCA
Pac02G011390R	TCACTTGCCGGGAACAAAGTT
Pac13G002370F	ATGGCAACAATGTTGAGCTCTGG
Pac13G002370R	TTATGAGCCAGGCGCAAACTT
Pac23G007730F	ATGACTGCGGCAACATCTGCCA
Pac23G007730R	TTACAGGCCAAGGGCACCAA
Pac17G010120F	ATGGCTGCATCTACAATCCAA
Pac17G010120R	TCACTTTCCAGGGACAAAGTT
Pac33G008510F	ATGGCTGCCTCTACAATCCAACAA
Pac33G008510R	TCACTTTCCAGGGACAAAG
For qPCR	
qEVM0007929F	GGAAGACTGCAGCCAAACCCAA
qEVM0007929R	ATGGGGACTCGCCTGAGAAT
qEVM0015298F	AACCGCCTCCGAGATCCTT
qEVM0015298R	CTTGACACGGTCTGGACCGTAC
qPac02G011390F	GCTTGGTCCATGCACAGAGCA
qPac02G011390R	ATAGAGTGGGTCGACCACCTCAC
q215.370.730F	TCTAGGGCAAGGTAGAGGAGCAA
q215.370.730R	CTGTCTGGCCCATACCACAAT
q62.120.510F	CAATCTGCATTTGCTGGGCAG
q62.120.510R	TTGAGGAGCACTCTTGACAGTGC

We also found that β-amylase genes in starch and sucrose metabolism were significantly down-regulated compared with other DEGs in the selected KEGG pathways in *P. americana*. An additional phylogenetic tree was constructed based on the deduced amino acid sequences for the β-amylase DEGs in *P. americana* and *P. acinosa* together with β-amylases with reviewed functions in UniProt (Hall and Notes, 2013). According to the reference genomes of *P. americana* and *P. acinosa*, candidate β-amylase genes were amplified with the primers listed in **Table 2**, and then sequenced. Substrate-binding and protein active sites of the deduced amino acid sequences from the amplified sequences were verified according to the homologous β-amylase in *Ipomoea batatas* (Yoshida and Nakamura, 1991) using SnapGene (4.2.4, GSL, Biotech, USA). qPCR was also conducted to analyze the β-amylase gene expression of the seedlings at 0, 1, 2, 3, and 4 DAG.

**Table 2:** Primers for β-amylase genes.

Name	Sequences 5’-3’
For β-amylase amplification	
244F	ATGGAATTGCCAGAGGAT
244R	TCACAAATTCCTTGAAAATC
61.00F	ATGGATAAGATGCTCTTGAA
61.00.17R	TTACCAACTGTCTACTTTCA
17F	ATGGGTTCAAATCTCATCAA
295F	ATGGATAAGATTGCTCTTGA
295R	TCAAGTGGAATGATTTTTCTTC
460F	ATGGATAAGATGCTCTTGAA
460R	TTACCAACTGTCTACTTTCA
610F	ATGCAAGTCCCAGCTAAA
610R	TTACCAACTGTCTACTTTCATGTC
300F	ATGGCGACCACAGGAGGG
300R	TCACTTAAACATGGAAAAAATCTTG
For qPCR of β-amylase	
PameqPCRF	GATGCAGGAGAACTAAGATACCCT
PameqPCRR	CAGCTGTTGCAGCTTTCTTGA
PaciqPCRF	AGAGTGGTCCACAACAACTGGT
PaciqPCRR	CCTAGCGTTCAACAGCATCTGA

### cDNA preparation and qPCR analysis

Emerged seedlings at 0, 1, 2, 3, and 4 DAG of the two species were separately collected and stored at -80°C after flash freezing in liquid nitrogen. Total RNAs were extracted as described above, and cDNA was subsequently prepared using 1.0 μg RNA using the FastQuant RT kit (Tiangen Biotech Co., Ltd, Beijing, China) according to the manufacturer’s instructions.

SnapGene was used to design the primers to quantify target gene expression (**Tables 1**, **2**). qRCR analysis was conducted using SuperReal PreMix Plus kits (SYBR Green; Tiangen Biotech Co., Ltd., Beijing, China) and an ABI QuantStudio 7 Flex PCR system (Applied Biosystems, Carlsbad, CA, USA). Samples were detected separately in 20 μL reaction mixture volumes with an initial thermal profile of 15 min at 95°C, followed by 40 cycles of 10 s at 95°C and 32 s at 60°C. Three technical replicates were performed for each sample, and three biological samples were used.

For qPCR analysis, we used the absolute quantitative method where the copy number of each gene was determined according to a standard curve. The standard curve was generated using a 10-fold serial dilution of standard plasmid ranging from 1 × 10^6^ to 1 × 10^2^ copies/μL target fragments (Liu et al., 2016).

### β-amylase activity and soluble sugar content in *P. americana* and *P. acinosa*

Emerged seedlings collected at 0, 1, 2, 3, and 4 DAG of the two species were also used to determine β-amylase activity and soluble sugar content. The β-amylase activity and soluble sugar content of each sample were separately determined following the instructions of β-amylase kits (Grace Biotechnology Co., Ltd. Suzhou, China) and soluble sugar content kits (Grace Biotechnology Co., Ltd. Suzhou, China) using a microplate reader (SuperMax 3100, Flash, China).

To validate the role photosynthesis played in the production of soluble sugars during seedling emergence, we cultured initial germinating seeds of the two species in the dark, and then collected emerged seedlings at 0, 1, 2, 3, and 4 DAG of the two species. Soluble sugar content of each sample was determined as described above, and three replicates were performed for each treatment.

### Data analysis

Data were analyzed using R. Student’s *t*-tests were used to compare germination rates, the number of emerged seedlings on the same day, and the fresh weight of the 10 days old seedlings between *P. americana* and *P. acinosa*. Likewise, expression levels of target genes, β-amylase activity, and soluble sugar content of the samples collected at the same time were also compared using *t*-tests. The number of emerged seedlings, expression level of target genes, β-amylase activity, and soluble sugar content of the same species at different times were analyzed using one-way analysis of variance (one-way ANOVA) and pair-wise *post hoc* analyses by least significant difference (LSD) in the agricolae package. Statistical significance was set at *P* < 0.05.

## Results

### Germination rate, seedling emergence, and seedling fresh weight for *P. americana* and *P. acinosa*

**Figure 1:**
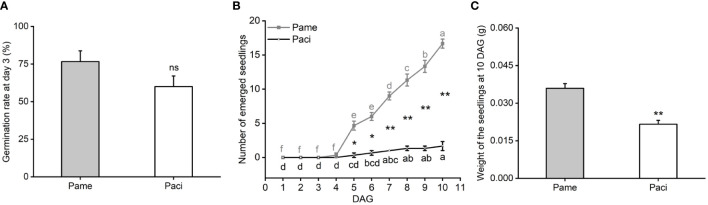
Germination rate at day 3 after seed dormancy breaking treatment **(A)**, number of emerged seedlings with spread cotyledons **(B)** and weight of the 10 DAG seedlings **(C)** of *Phytolacca americana* (Pame) and *Phytolacca acinosa* (Paci). DAG: day(s) after germination. Values represent the mean ± standard error. Different letters in the same shade as the line indicate that the means differ significantly among the tested time points under one-way ANOVA followed by least significant difference (LSD) test. Asterisks indicate a significant difference between the two species at the same time point under Student’s *t*-test. *P < 0.05, **P < 0.01, ns *P* > 0.05.

After being treated with H_2_SO_4_ to break seed dormancy, we found *P. americana* and *P. acinosa* seeds began to germinate at day 3, and their germination rates were not significantly different (*t*-test, *P* = 0.1152; **Figure 1A**). These germinated seeds were then used for the following experiments.

After culturing the initially germinated seeds for 10 days, we found that *P. americana* seedlings began to emerge at 4 DAG (one-way ANOVA, *P*_Pame_ < 0.001), while *P. acinosa* began to emerge at 5 DAG (one-way ANOVA, *P*_Paci_ < 0.001). At 10 DAG, approximately 80% of the tested *P. americana* seeds developed into seedlings, while less than 10% of the *P. acinosa* seedlings developed. Additionally, the number of emerged *P. americana* seedlings was significantly higher than that of *P. acinosa* (*t*-test; *P*_5 DAG_ = 0.004; *P*_6 DAG_ = 0.001; *P*_7 DAG_ < 0.001; *P*_8 DAG_ < 0.001; *P*_9 DAG_ < 0.001; *P*_10 DAG_ < 0.001; **Figure 1B**). At 10 DAG, the fresh weight of *P. americana* seedlings was also significantly higher than that of *P. acinosa* seedlings (*t*-test, *P* = 0.004; **Figure 1C**). Together, these results indicate that the time intervals between seed germination and seedling emergence of invasive *P. americana* were shorter than that of native *P. acinosa*.

### Genome-wide transcriptome data analysis

**Figure 2:**
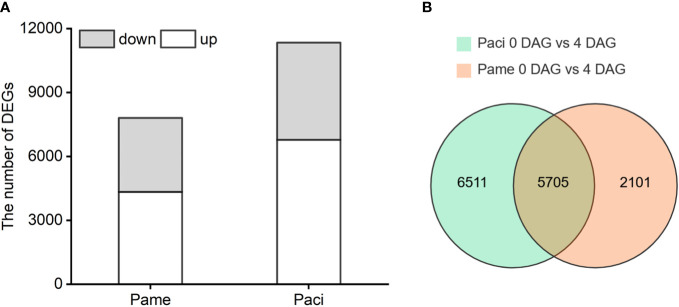
Differentially expressed genes (DEGs) of the 0 vs 4 DAG seedlings of *Phytolacca americana* (Pame) and *Phytolacca acinosa* (Paci) **(A)**, and homologous DEGs between 0 vs 4 DAG seedlings of *P. americana* and *P. acinosa* **(B)**. DAG: day(s) after germination. Grey represents the number of down-regulated DEGs, and white represents the number of up-regulated DEGs.

RNA-Seq data of the tested samples were evaluated using Pearson’s correlation coefficient (r) and showed high consistency (r > 0.94) within groups (**Supplementary Figure S2**). The average FPKM of each group was then used for DEG analysis. In total, 7,806 and 11,340 DEGs were identified in the 0 vs 4 DAG seedlings of *P. americana* and *P. acinosa*, respectively. Of these, 4,341 and 3,465 genes in *P. americana*, and 6,779 and 4,561 genes in *P. acinosa* were up- and down-regulated, respectively (**Figure 2A**). In both *P. americana* and *P. acinosa*, 5,705 DEGs showed more than 50% similarity and were treated as the intersection of the Venn diagram (**Figure 2B**; **Supplementary Material**). We also found 2,101 DEGs only in the 0 vs 4 DAG seedlings for *P. americana*, and 6,511 DEGs only in the 0 vs 4 DAG seedlings for *P. acinosa*.

According to the annotations on the GO database, *P. americana* seedling DEGs were highly enriched in the metabolic and cellular processes in the biological process category, cell and cell part in the cellular component category, as well as binding and catalytic activity in the molecular function category. The same trend was also observed in *P. acinosa* seedlings. However, the number of DEGs in *P. acinosa* was higher than that in *P. americana* (**Supplementary Figure S3**).

KEGG pathway enrichment analysis suggested that 26 pathways were differentially enriched by the DEGs in *P. americana*, while 19 pathways were enriched in *P. acinosa* (**Supplementary Figure S4**). Among these KEGG pathways, 12 pathways were only found in *P. americana* seedlings, while only five were in *P. acinosa* seedlings. Furthermore, energy metabolism pathways, such as butanonate metabolism, pentose phosphate pathway, and starch and sucrose metabolism, were only found in *P. americana*. According to the FPKM values, DEGs for the photosynthesis-antenna proteins pathway in both species were highly up-regulated. However, some DEGs for starch and sucrose metabolism were significantly down-regulated in *P. americana* (**Supplementary Figures S5, S6**).

### DEGs in photosynthesis-antenna proteins pathway

**Figure 3:**
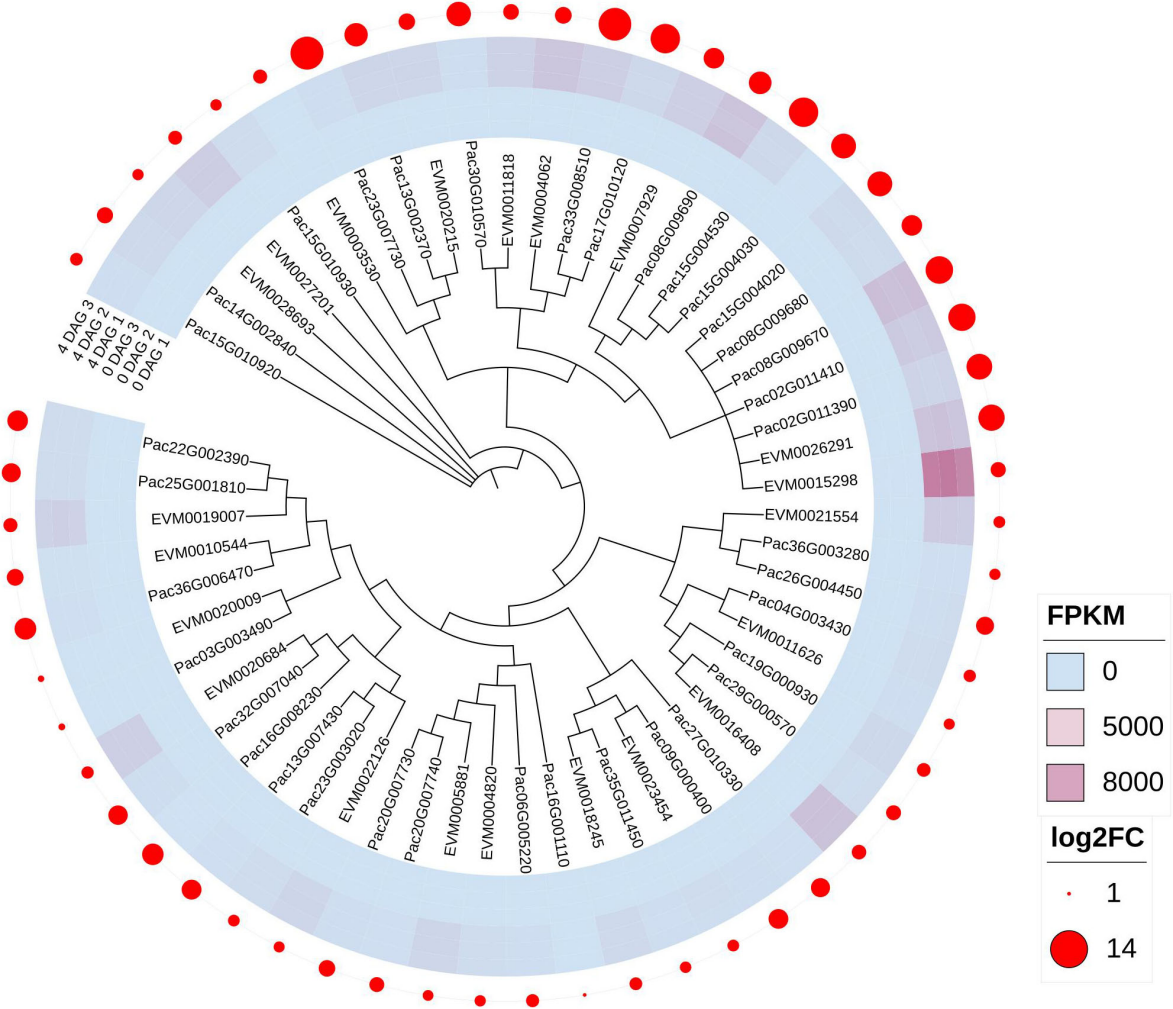
Differentially expressed genes (DEGs) enriched in the photosynthesis-antenna proteins pathway between *Phytolacca americana* and *Phytolacca acinosa*. DAG: day(s) after germination. FPKM represent the fragment per kilobase of exon model per million mapped reads value of each DEG, and log_2_FC is the log2foldchange (0 DAG versus 4 DAG) of each DEG. EVM is the DEG in *P. americana*, and Pac is the DEG in *P. acinosa*.

In total, we filtered 23 photosynthesis-antenna proteins pathway DEGs in the 0 vs 4 DAG seedlings for *P. americana* and 37 DEGs in *P. acinosa* seedlings. The phylogenetic tree suggested that photosynthesis-antenna proteins pathway DEGs between *P. americana* and *P. acinosa* were highly similar. Even though these DEGs were significantly up-regulated, many DEGs in *P. acinosa* seedlings were more highly expressed compared to their homogeneous genes in *P. americana* (**Figure 3**).

**Figure 4:**
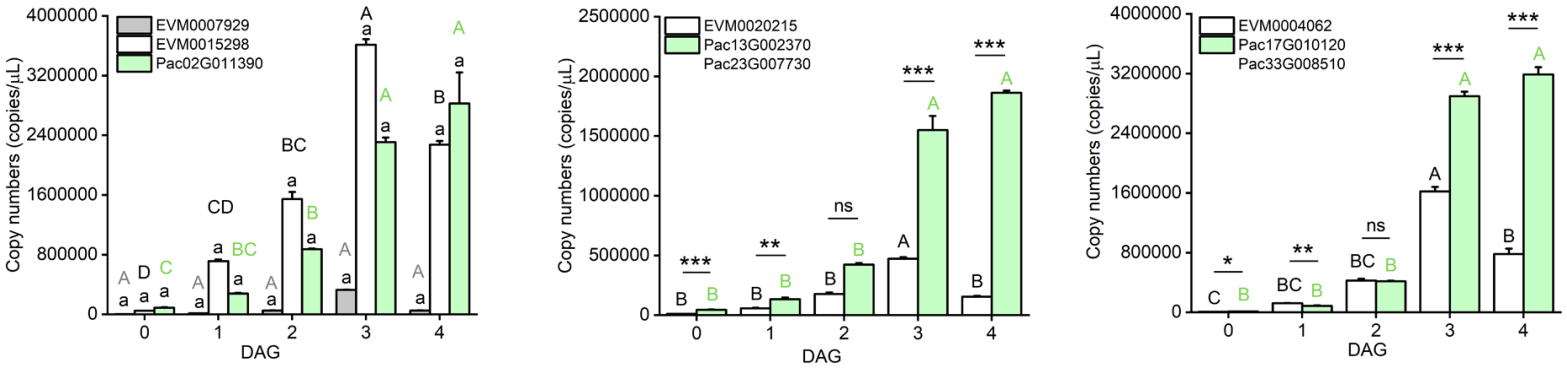
Expression level of homologous fragments of genes involved in photosynthesis-antenna proteins pathway between *Phytolacca americana* and *Phytolacca acinosa*. DAG: day(s) after germination. Values represent the mean ± standard error. Different letters in the same color as the column indicate that the means differ significantly among the tested time points under one-way ANOVA followed by least significant difference (LSD) test. Asterisks indicate a significant difference between the two species at the same time point under Student’s *t*-test. *P < 0.05, **P < 0.01, ***P < 0.001, ns *P* > 0.05.

Additionally, we compared the gene expression among seedlings collected at 0, 1, 2, 3, and 4 DAG, and found that the tested DEGs in *P. americana* had the highest expression at 3 DAG, while in *P. acinosa* they showed the highest expression at 4 DAG (**Figure 4**).

### Candidate genes of β-amylase

**Figure 5:**
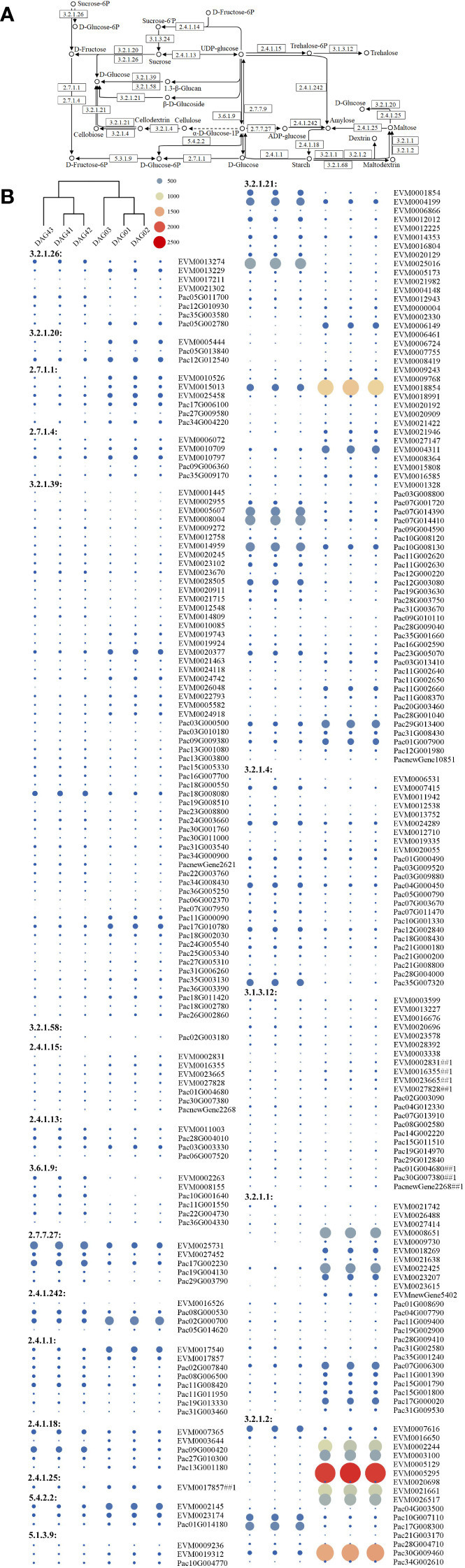
Starch and sucrose metabolism pathway and the related differentially expressed genes (DEGs) in *Phytolacca americana* and *Phytolacca acinosa*. **(A)** Starch and sucrose metabolism pathway that DEGs were detected in *P. americana* and *P. acinosa*. **(B)** Heatmap of the DEGs based on the fragment per kilobase of exon model per million mapped reads values. DAG: day(s) after germination. ## indicates the sequence is related to more than one enzyme. DAG01, DAG02, and DAG03 are the three replicates of 0 DAG seedlings, while DAG41, DAG42, and DAG43 are the three replicates of 4 DAG seedlings.

**Figure 6:**
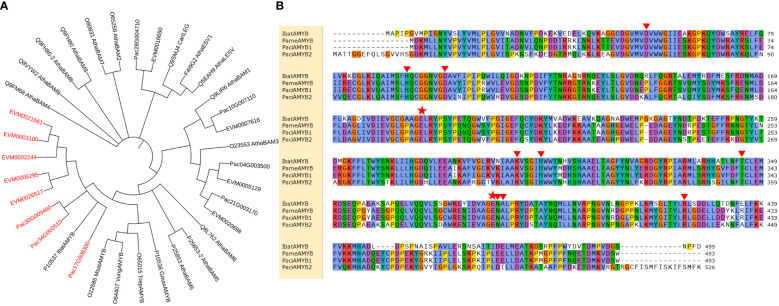
β-amylase in *Phytolacca americana* and *Phytolacca acinosa*. **(A)** Phylogenetic tree of β-amylase in *P. americana* and *P. acinosa* together with other reported β-amylases in Uniprot. **(B)** Sequence alignment of β-amylase in *P. americana* (PameAMYB), *P. acinosa* (PaciAMYB1 and PaciAMYB2), and *Ipomoea batatas* (IbatAMYB). EVM is the DEG in *P. americana*, and Pac is the DEG in *P. acinosa*. Red font represents the target sequences. Red triangles indicate the substrate binding sites of the proteins, and red pentangles show the active sites of the proteins.

We then selected and compared DEGs involved in the synthesis of 21 enzymes for starch and sucrose metabolism. Genes for β-glucosidase and β-amylase in *P. americana* were differentially expressed during the first four days after germination (**Figure 5**). The phylogenetic tree of β-amylase in *P. americana* and *P. acinosa* together with other plants reported in UniProt were constructed and showed that five DEGs for β-amylases in *P. americana* and three DEGs in *P. acinosa* were clustered with β-amylase in *I. batatas* (IbatAMYB; **Figure 6A**).

Further PCR amplification and sequence alignment revealed that four of the five β-amylase genes in *P. americana* were amplified and showed the consensus sequence (named *PameAMYB*). However, the remaining transcript could not be amplified. Additionally, two of the three transcripts for β-amylase in *P. acinosa* were confirmed to be the same and were named *PaciAMYB*1 and *PaciAMYB*2. Further, sequence alignment of IbatAMYB, PameAMYB, PaciAMYB1 and PaciAMYB2 suggested that all the binding and active sites were present among the deduced amino acid sequences (**Figure 6B**). Together, these results suggest the presence of functional β-amylase in both *P. americana* and *P. acinosa*.

### Gene expression and enzymatic activity of β-amylase

**Figure 7:**
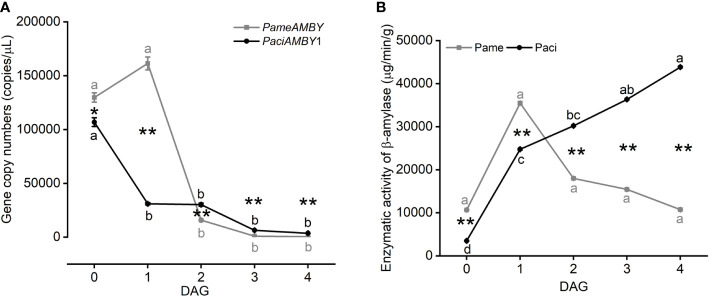
Gene expression of *PameAMBY* and *PaciAMBY*1 **(A)**, and enzymatic activity of β-amylase **(B)** among samples of *Phytolacca americana* (Pame) and *Phytolacca acinosa* (Paci). DAG: day(s) after germination. Values represent the mean ± standard error. Different letters in the same shade as the line indicate significantly different means of the species under one-way ANOVA followed by least significant difference (LSD) test. Asterisks indicate a significant difference between the two species at the same time point under Student’s *t*-test. *P < 0.05, **P < 0.01.

At 0 DAG, β-amylase gene expression was high for both species. *PameAMYB* expression was higher in *P. americana* at 1 DAG, but then sharply decreased (one-way ANOVA, *P*_Pame_ < 0.001; **Figure 7A**). In comparison, *PaciAMYB*1 decreased from 0 DAG (one-way ANOVA, *P*_Paci_ < 0.001). Although the gene expression level of *PameAMYB* was lower at 2, 3, and 4 DAG compared to *PaciAMYB*1, *PameAMYB* was highly expressed at 0 and 1 DAG (*t*-test, *P*_0 DAG_ = 0.018, *P*_1 DAG_ < 0.001, *P*_2 DAG_ = 0.001, *P*_3 DAG_ < 0.001, *P*_4 DAG_ < 0.001).

The enzymatic activity measurements suggested that β-amylase activity was the highest at 1 DAG in *P. americana* and decreased at 4 DAG to a level similar to 0 DAG. However, β-amylase activity was not significantly different during those four days (one-way ANOVA, *P*_Pame_ = 0.265; **Figure 7B**). In *P. acinosa*, the β-amylase activity gradually increased (one-way ANOVA, *P*_Paci_ < 0.001) and began to be higher than *P. americana* at 2 DAG (*t*-test, *P*_0 DAG_ < 0.001, *P*_1 DAG_ < 0.001, *P*_2 DAG_ < 0.001, *P*_3 DAG_ < 0.001, *P*_4 DAG_ < 0.001), and this trend could be related to the expression of *PaciAMBY*2, since it was up-regulated at 0 vs 4 DAG in *P. acinosa* (log_2_FC of *PaciAMBY*2 = 4.75, **Supplementary Material**).

### Total soluble sugars determination

**Figure 8:**
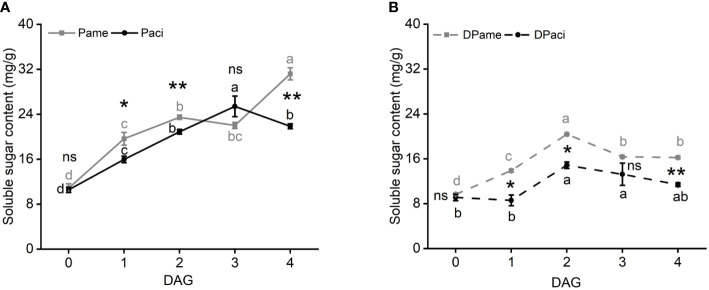
Total soluble sugar content among samples of *Phytolacca americana* (Pame) and *Phytolacca acinosa* (Paci) cultured in light/dark cycle **(A)** or in the dark **(B)**. Pame: *P. americana* seedlings are cultured in the light/dark cycle; Paci: *P. acinosa* seedlings are cultured in the light/dark cycle. DPame: *P. americana* seedlings are cultured in the dark; DPaci: *P. acinosa* seedlings are cultured in the dark. Values represent the mean ± standard error. Different letters in the same shade as the line indicate significantly different means of the species under one-way ANOVA followed by least significant difference (LSD) test. Asterisks indicate a significant difference between the two species at the same time point under Student’s *t*-test. *P < 0.05, **P < 0.01, ns *P* > 0.05.

Total soluble sugar content in *P. americana* increased in the first two days after germination, and reached its highest level at 4 DAG (one-way ANOVA, *P*_Pame_ < 0.001; **Figure 8A**). However, total soluble sugars in *P. acinosa* increased from 0 to 3 DAG, but then decreased (one-way ANOVA, *P*_Paci_ < 0.001). At 0 DAG, the soluble sugar content was not significantly different between the two species (*t*-test, *P* = 0.748), and was higher at 1 (*t*-test, *P* = 0.045) and 2 (*t*-test, *P* = 0.009) DAG in *P. americana* compared to *P. acinosa*, but then decreased at 3 DAG (*t*-test, *P* = 0.149). However, it was markedly higher in *P. americana* than in *P. acinosa* at 4 DAG (*t*-test, *P* = 0.001).

When the initially germinated seeds were cultured in the dark, the two species showed a similar variation in total soluble sugar content (one-way ANOVA; *P*_Pame_ < 0.001; *P*_Paci_ = 0.035), even though the sugar content in *P. americana* was higher than that in *P. acinosa* (*t*-test, *P*_0 DAG_ = 0.443, *P*_1 DAG_ = 0.034, *P*_2 DAG_ = 0.011, *P*_3 DAG_ = 0.259, *P*_4 DAG_ = 0.006). Moreover, soluble sugar content in *P. americana* did not significantly increase at 4 DAG (**Figure 8B**).

The germinated seeds cultured in the light-dark cycle also showed higher total soluble sugar content compared to those cultured in the dark (*t*-test, *P. americana*: *P*_0 DAG_ = 0.325, *P*_1 DAG_ = 0.030, *P*_2 DAG_ = 0.007, *P*_3 DAG_ = 0.004, *P*_4 DAG_ = 0.002; *P. acinosa*: *P*_0 DAG_ = 0.146, *P*_1 DAG_ = 0.006, *P*_2 DAG_ = 0.003, *P*_3 DAG_ = 0.022, *P*_4 DAG_ = 0.001). These results suggest that photosynthesis is vital for the formation of soluble sugars in *P. americana* at 4 DAG.

## Discussion

### Invasive *P. americana* shows faster seedling emergence compared to native *P. acinosa*

It has been reported that invasive plants show faster seedling emergences compared to native ones, such as *Bromus tectorum* (Griffith et al., 2014), *Sorghum halepense* (Reichmann et al., 2016), and *Spartina densiflora* (Infante-Izquierdo et al., 2020). In our study, even though *P. americana* and *P. acinosa* seeds began to germinate at the same time and showed similar germination rates, the invasive *P. americana* seedlings emerged more rapidly than native *P. acinosa* (**Figures 1A, B**). Typically, emerged seedlings use resources for growth and, therefore, gain competitive ability. Under resource competition, early seedling emergence could lead to disproportional resource utilization and create fecundity advantages (Dyer et al., 2000). We found that *P. americana* showed seedling emergence one day earlier than *P. acinosa*, which suggest that invasive *P. americana* could utilize resources earlier than native *P. acinosa*. Ross and Harper (1972) reported that 95% of plant biomass variation could be accounted for by time since emergence. Here, the biomass of *P. americana* seedlings at 10 DAG was significantly higher than *P. acinosa* seedlings (**Figure 1C**). This is also similar to the previous data that *P. americana* had a higher total biomass and exhibited a higher reproductive capacity compared to *P. acinosa* (Liu et al., 2022). Additionally, to avoid the effect of seed batch on seed germination and seedling emergence, we compared the data among seeds collected in 2019, 2021 to 2023, and found similar data among one particular species during the years (**Supplementary Figures S7, S8**).

Our transcriptome data also showed that many genes were differentially expressed at 0 vs 4 DAG in both *P. americana* and *P. acinosa* seedlings. Total DEGs in *P. acinosa* were higher than in *P. americana* (**Figure 2**), which may be related to the ploidy level of these plants, since *P. americana* is diploid and *P. acinosa* is autotetraploid. Similar results were found in autotetraploid rice and its diploid donor (Wang et al., 2022). GO analysis of the 0 vs 4 DAG seedlings of *P. americana* and *P. acinosa* also suggest that the two species undergo similar development (**Figure 3**). However, the KEGG pathway enrichment analysis suggests that the differential KEGG pathways between the two congeners are not entirely consistent (**Supplementary Figure S3**). More pathways are activated in invasive *P. americana* than in native *P. acinosa*, especially pathways related to energy metabolism. Studies found that genes in slowly and rapidly developing *Panicum virgatum* are differentially expressed in pathways related to diterpenoid biosynthesis, thiamine metabolism, and circadian rhythm (Zhang et al., 2019). The inconsistent results might be caused by the varying developmental stages of tested samples.

### Photosynthesis and β-amylase play important roles in *P. americana*’s rapid seedling emergence

High photosynthetic rates in the aerial parts of invasive *Mikania micrantha* contribute to its rapid growth (Liu et al., 2020). Additionally, the rapid formation of the photosynthetic system promotes post-germination growth of *Pennisetum glaucum* (Wu et al., 2021), and is highly dependent on cotyledon photosynthesis (Zhang et al., 2008). In our study, photosynthesis related pathways are activated in the emerged seedlings from both *P. americana* and *P. acinosa*, and the induced genes involved in these photosynthesis-antenna proteins pathways share high similarities in the two species (**Figure 3**). However, photosynthesis-related genes show the highest expression at 3 DAG in *P. americana*, but are the highest at 4 DAG in *P. acinosa* (**Figure 4**), which may facilitate the seedling emergence of *P. americana*.

In addition, amylase genes are positively correlated with invasion success of *Dendroctonus valens* (Liu F. et al., 2020). In our study, β-amylase genes in starch and sucrose metabolism are differentially expressed at 0 vs 4 DAG seedlings of *P. americana* (**Figure 5**). Although β-amylase plays a minor role in storage starch degradation in wheat (Kaneko et al., 2000; Sun et al., 2020), β-amylase activity in *P. americana* is the highest at 1 DAG, which is in accordance with its gene expression level of *PameAMYB*, and continuous substrate catalysis leads to the accumulation of soluble sugars, and a soluble sugar content decrease at 3 DAG with increasing consumption during growth (**Figures 7**, **8**). However, soluble sugars could also be formed by the photosynthetic system in the following days, as evidenced by the low soluble sugar content in emerged *P. americana* seedlings grown in the dark (**Figure 8**). Therefore, our data indicate that high soluble sugars produced by starch metabolism and photosynthesis contributes to *P. americana* rapid seedling emergence. Still, the role of *PameAMYB* in rapid seedling emergence of invasive *P. americana* should be further tested using genome editing in the future.

In conclusion, our results show that invasive *P. americana* seedlings emerge faster than native *P. acinosa*. KEGG pathway analysis of 0 vs 4 DAG seedlings suggested that DEGs in photosynthesis-antenna proteins pathway were up-regulated in both *P. americana* and *P. acinosa*, while DEGs in starch and sucrose metabolism were significantly down-regulated in *P. americana*. Gene expression analysis indicated that photosynthesis-related DEGs in *P. americana* seedlings reached the highest level at 3 DAG, while *P. acinosa* seedlings showed their peak at 4 DAG. We also identified one β-amylase gene in *P. americana* (*PameAMYB*) that showed the highest expression at 1 DAG, and two β-amylase genes in *P. acinosa* that *PaciAMYB*1 expressed the highest at 0 DAG, while *PaciAMYB*2 at 4 DAG. However, at 0 DAG, the expression of *PaciAMYB*1 was lower than *PameAMYB*. Enzymatic activity of β-amylases determination suggested that *P. americana* had the highest β-amylases activity at 1 DAG, which was earlier than in *P. acinosa* (at 4 DAG). Soluble sugars, the main source of energy for seedling emergence, were showed higher in *P. americana* than in *P. acinosa*, and reached the highest at 4 DAG. Moreover, soluble sugar content was significantly decreased at 4 DAG in *P. americana* when cultured the germinated seeds in the dark. These results suggest that high soluble sugar content in *P. americana* produced by starch metabolism and photosynthesis benefits the rapid seedling emergence, enhancing our knowledge on physiological mechanisms of invasion success.

## Data availability statement

The original data presented in the study are included in the article/**Supplementary Material**. Further inquiries can be directed to the corresponding author.

## Author contributions

DL: Conceptualization, Funding acquisition, Investigation, Software, Validation, Writing – original draft, Writing – review & editing. ML: Data curation, Investigation, Writing – original draft. RJ: Funding acquisition, Writing – review & editing. BL: Funding acquisition, Writing – review & editing. YW: Funding acquisition, Supervision, Writing – review & editing.
