# Supplementary material for: Rapid seedling emergence of invasive Phytolacca americana is related to higher soluble sugars produced by starch metabolism and photosynthesis compared to native P. acinosa
Source: Front Plant Sci. 2024 Jan 26;15:1255698. doi: 10.3389/fpls.2024.1255698 (PMC10853419; doi:10.3389/fpls.2024.1255698)
Supplement: Supplementary file 1 [file Image_1.pdf]

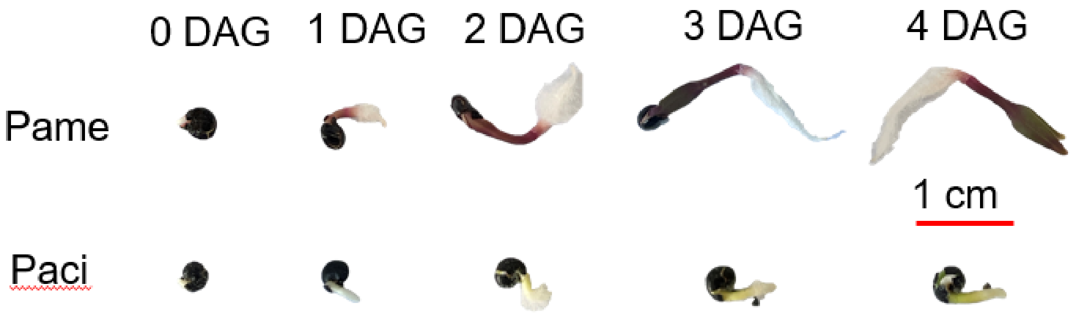

Figure S1 Samples collected on the first 4 days after seed germination. Pame: *Phytolacca americana*, Paci: *Phytolacca acinosa*. DAG: day(s) after germination.

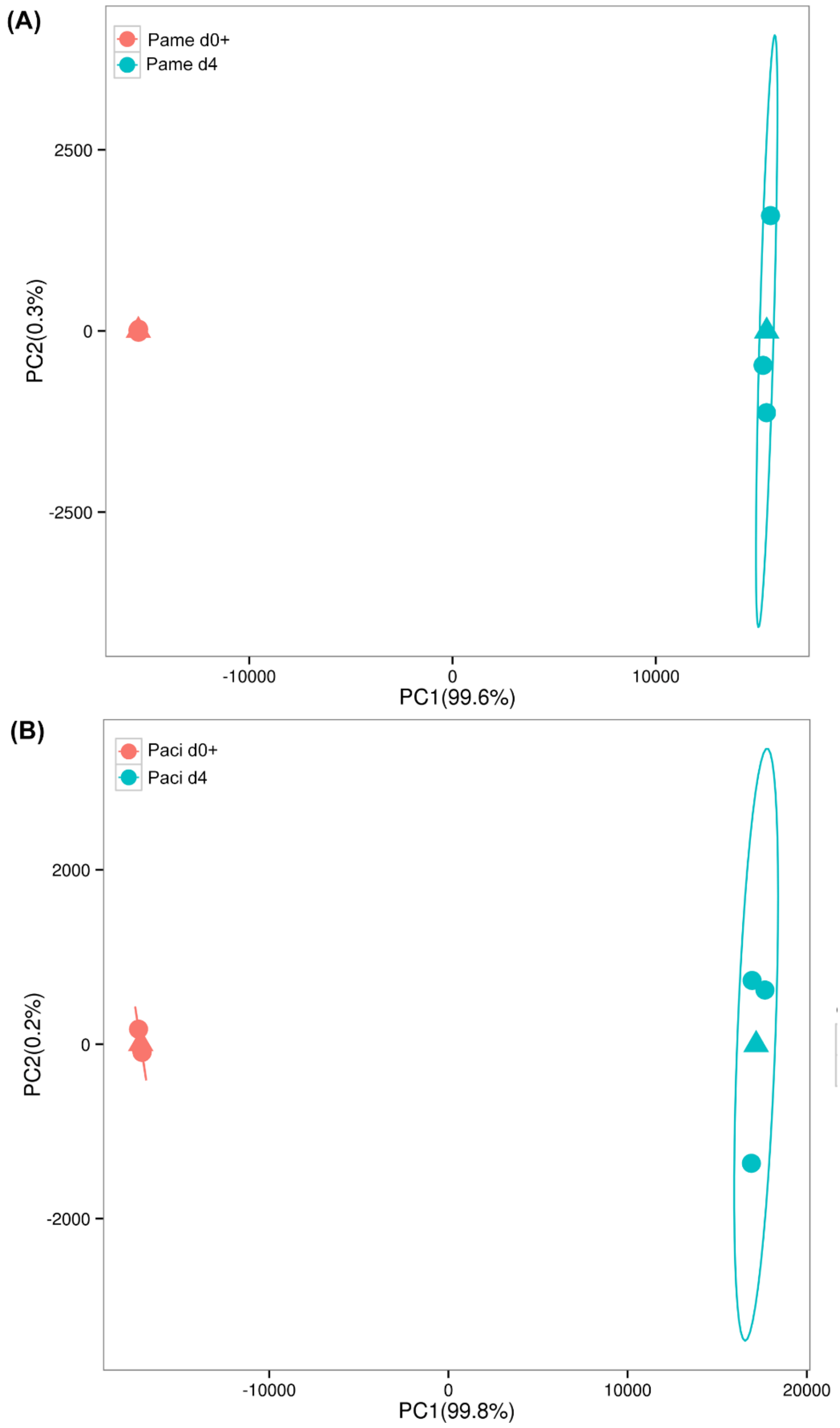

Figure S2 Principal component analysis plots of transcripts identified by RNA-Seq of *Phytolacca americana* (A) and *Phytolacca acinosa* (B) 0 vs 4 DAG seedlings. DAG: day(s) after germination.

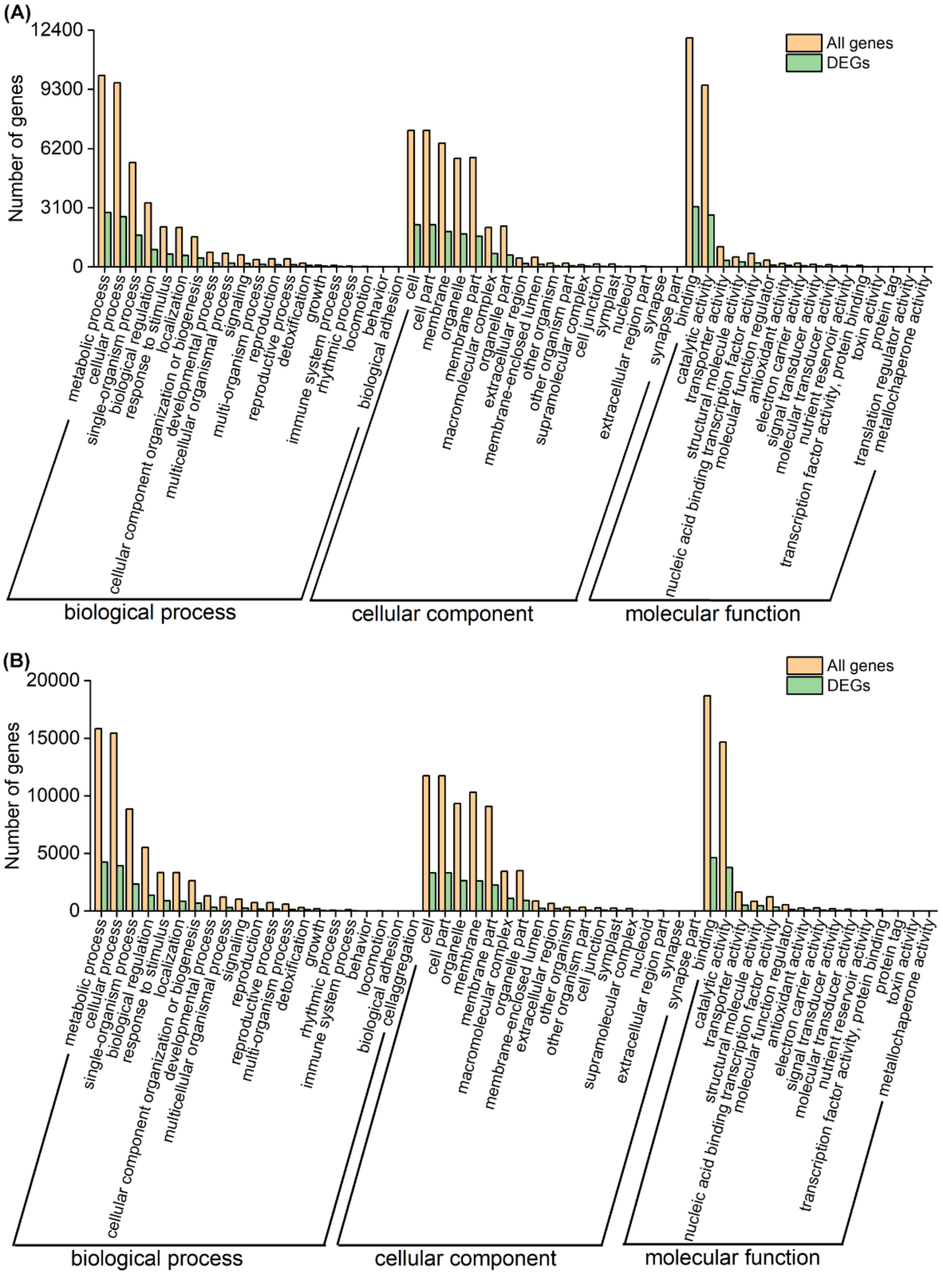

Figure S3 Gene Ontology enrichment analysis of all detected genes and differentially expressed genes (DEGs) in initially germinated seeds vs 4 days after germination seedlings of *Phytolacca americana* (A) and *Phytolacca acinosa* (B). Orange columns show the number of all detected genes, and green columns represent the number of DEGs.

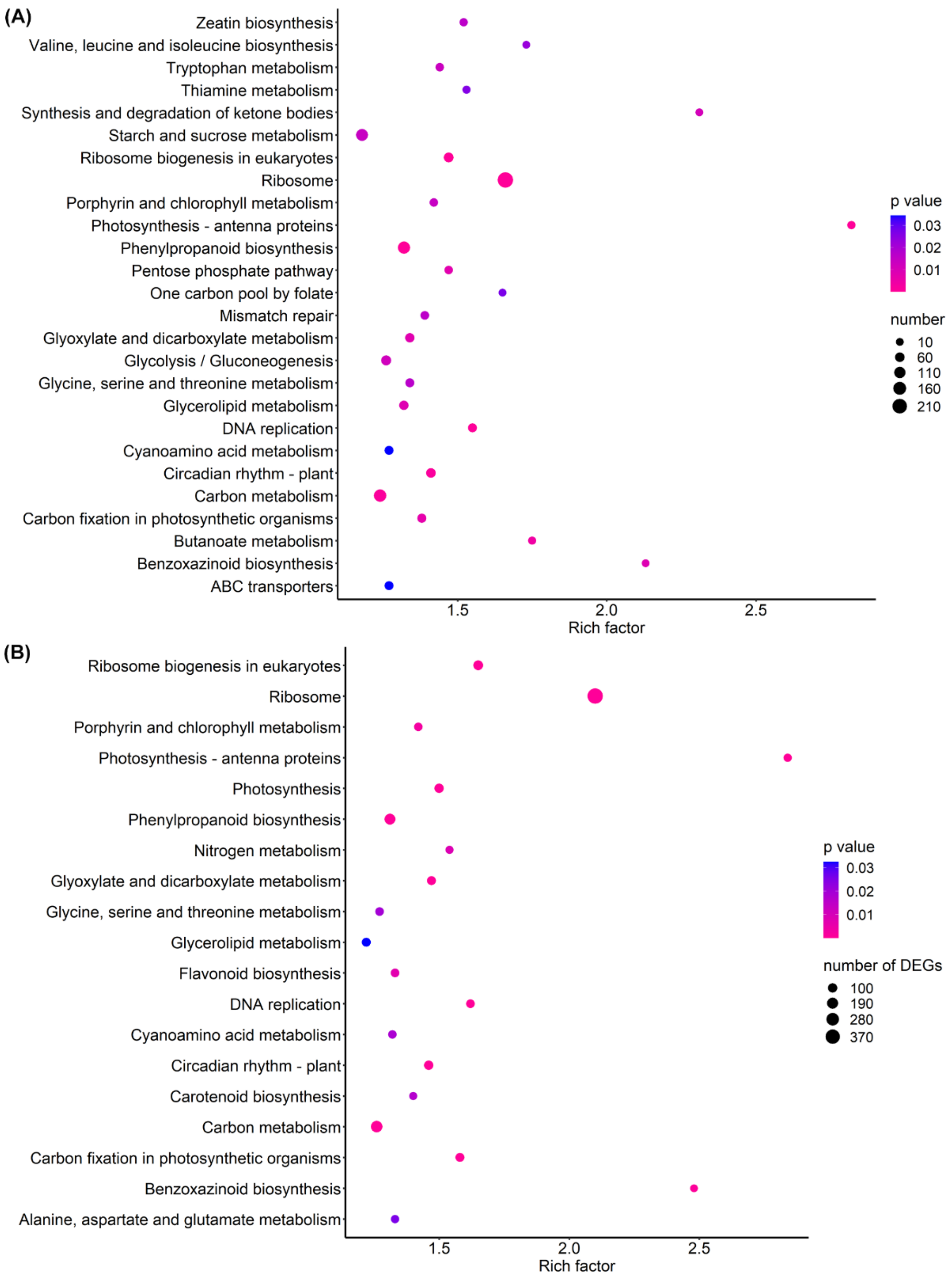

Figure S4 Metabolism pathways that were significantly enriched by the differentially expressed genes.

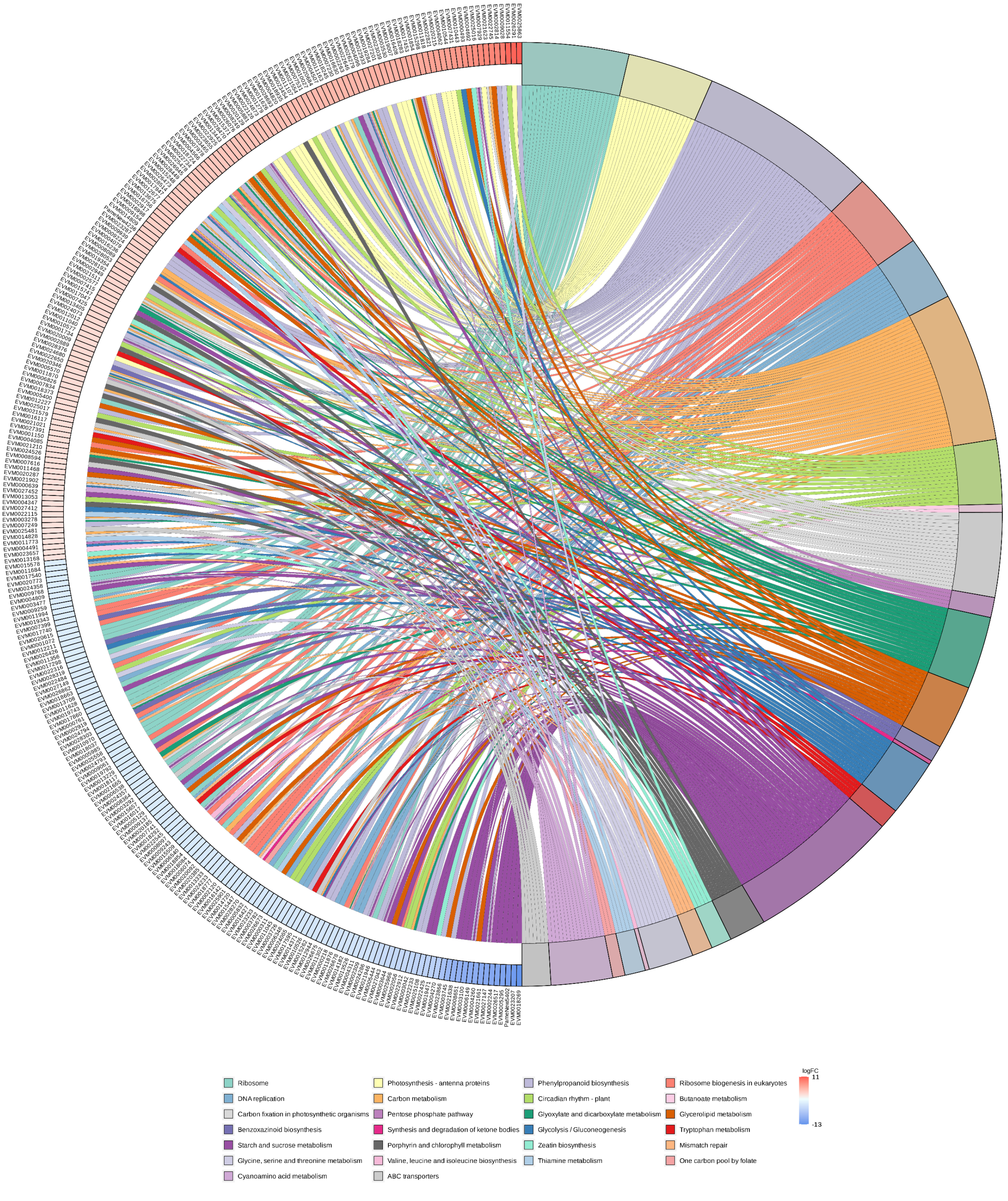

Figure S5 Metabolism pathways and the differentially expressed genes in 0 vs 4 DAG seedlings of *Phytolacca americana*. DAG: day(s) after germination.

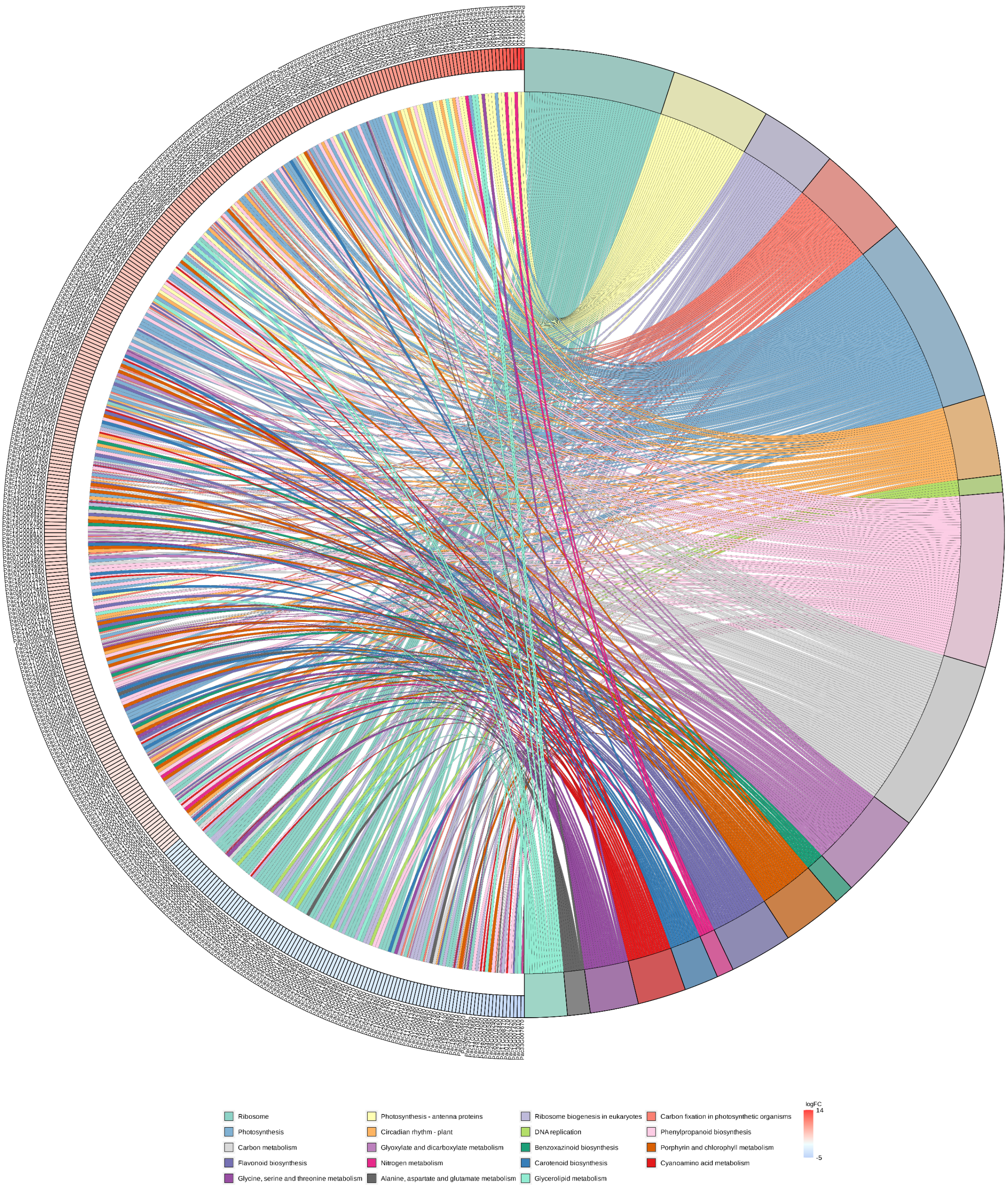

Figure S6 Metabolism pathways and the differentially expressed genes in 0 vs 4 DAG seedlings of *Phytolacca acinosa*. DAG: day(s) after germination.

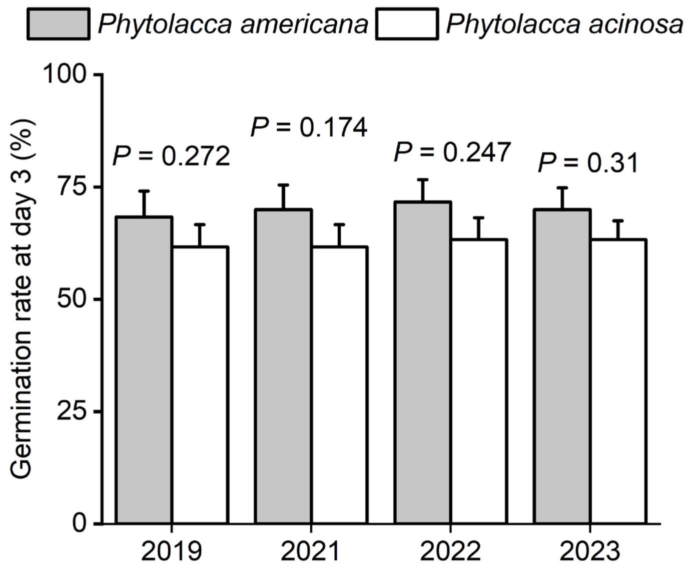

Figure S7 Germination rate at day 3 of *Phytolacca americana* and *Phytolacca acinosa* seeds collected in different years. The seeds of *P. americana* (24°49′ N, 102°52′ E) and *P. acinosa* (25°26′ N, 104°19′ E) were collected on August each year from 2019 to 2022. The seeds of the two species that collected in 2022 were cultured in a common garden (24°49′ N, 102°50′ E) in Yunnan University, and the seeds used in 2023 were collected from the common garden. The methods for seed germination were the same as described in Materials and Methods Section. The germination rate of *P. americana* and *P. acinosa* were analyzed under Student's *t*-test.

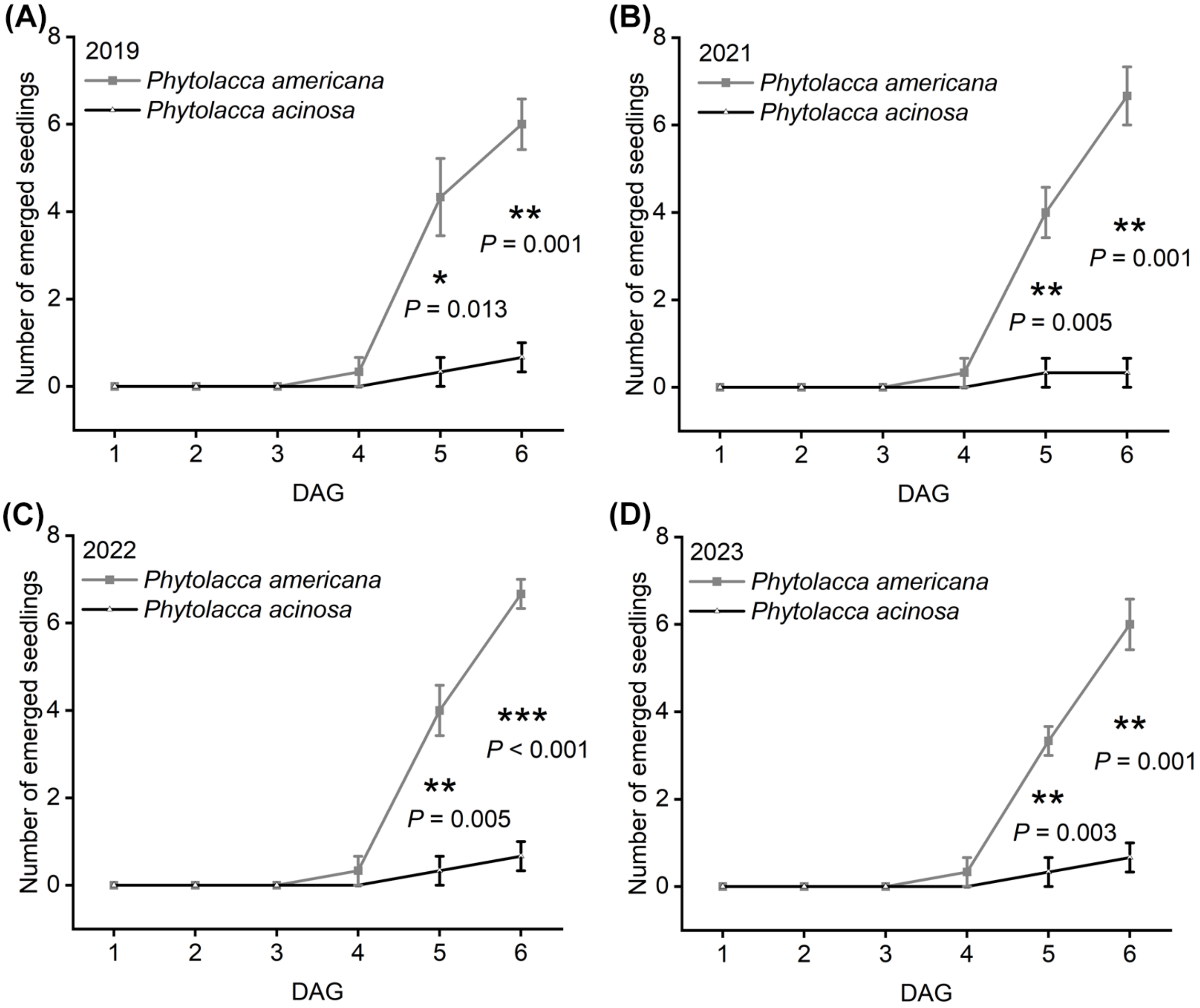

Figure S8 Number of emerged seedlings of *Phytolacca americana* and *Phytolacca acinosa* within 6 days after germination (DAG). The seeds used were the same as described in Figure S7, and the methods for seedling emergence were the same as described in Materials and Methods Section. Asterisks indicate a significant difference between the two species at the same time point under Student's *t*-test. * $P < 0.05$, ** $P < 0.01$, *** $P < 0.001$.
